# Preference of a Polyphagous Mirid Bug, *Apolygus lucorum* (Meyer-Dür) for Flowering Host Plants

**DOI:** 10.1371/journal.pone.0068980

**Published:** 2013-07-10

**Authors:** Hongsheng Pan, Yanhui Lu, Kris A. G. Wyckhuys, Kongming Wu

**Affiliations:** 1 State Key Laboratory for Biology of Plant Diseases and Insect Pests, Institute of Plant Protection, Chinese Academy of Agricultural Sciences, Beijing, China; 2 International Center for Tropical Agriculture CIAT-Asia, Hanoi, Vietnam; Volcani Center, Israel

## Abstract

*Apolygus lucorum* (Meyer-Dür) (Hemiptera: Miridae) is one of the most important herbivores in a broad range of cultivated plants, including cotton, cereals, vegetables, and fruit crops in China. In this manuscript, we report on a 6-year long study in which (adult) *A. lucorum* abundance was recorded on 174 plant species from 39 families from early July to mid-September. Through the study period per year, the proportion of flowering plants exploited by adult *A. lucorum* was significantly greater than that of non-flowering plants. For a given plant species, *A. lucorum* adults reached peak abundance at the flowering stage, when the plant had the greatest attraction to the adults. More specifically, mean adult abundance on 26 species of major host plants and their relative standard attraction were 10.3–28.9 times and 9.3–19.5 times higher at flowering stage than during non-flowering periods, respectively. Among all the tested species, *A. lucorum* adults switched food plants according to the succession of flowering plant species. In early July, *A. lucorum* adults preferred some plant species in bloom, such as *Vigna radiata*, *Gossypium hirsutum*, *Helianthus annuus* and *Chrysanthemum coronarium*; since late July, adults dispersed into other flowering hosts (e.g. *Ricinus communis*, *Impatiens balsamina*, *Humulus scandens*, *Ocimum basilicum*, *Agastache rugosus* and *Coriandrum sativum*); in early September, they largely migrated to flowering *Artemisia* spp. (e.g. *A. argyi*, *A. lavandulaefolia*, *A. annua* and *A. scoparia*). Our findings underscore the important role of flowering plays in the population dynamics and inter-plant migration of this mirid bug. Also, our work helps understand evolutionary aspects of host plant use in polyphagous insects such as *A. lucorum*, and provides baseline information for the development of sustainable management strategies of this key agricultural pest.

## Introduction

Agricultural landscapes regularly consist of crop fields interspersed with uncultivated habitats, thus providing abundant food resources for generalist phytophagous insects [1], [2]. Change in the phenology of certain host or food plants results in a constantly changing mosaic of habitats across the agro-landscape [1], [3]. Most polyphagous plant-feeding insects ephemerally exploit suitable host plants and habitats, but equally engage in host plant switching to locate new, more suitable hosts [1], [4], [5]. One advantage of such periodic host switching is that it permits continuous exploitation of a nutrient-diverse diet, thereby improving survival and reproduction [1], [6], [7]. Additionally, polyphagous insect herbivores usually exhibit clear preferences for particular plant species or plant growth stages [7], [8], [9], [10], [11]. An in-depth assessment of host plant preferences of polyphagous insects is central to understanding their seasonal dynamics on a particular plant species and their movement between plants and habitats across the agricultural landscape.

Many polyphagous insects, such as butterflies and moths (Lepidoptera), show great preference for flowers [7], [12], [13], [14]. Many species of mirid bugs (Heteroptera: Miridae) prefer to feed on the relatively energy-rich plant tissues in flowers and buds [15], giving this insect group the common name “flower bugs” [16]. For example, *Lygus lineolaris* (Palisot de Beauvois) typically feeds on leaf buds and reproductive structures such as flower buds and flowers [17]. This mirid bug usually tracks a succession of flowering plant species, with plant colonization initiating at the formation of floral buds or flowers [18], and maximum abundance attained during bloom [19]. *Lygus hesperus* Knight often attained its peak of adult abundance in alfalfa, when that crop was in the blooming stage [11]. Similar phenomena already have been described in many other mirid bugs [15].

The mirid bug *Apolygus lucorum* (Meyer-Dür) (Hemiptera: Miridae) has historically been regarded as a minor pest in cotton and many other crops in China [20], [21]. However, the widespread adoption of transgenic Bt (*Bacillus thuringiensis*) cotton and subsequent reduction of insecticide sprays in Bt cotton has allowed *A. lucorum* to reach outbreak levels in cotton and several other agricultural crops [22]. *A. lucorum* adults and nymphs feed on vegetative and reproductive tissues of their host plants, causing stunted growth and the abscission or malformation of leaves, flowers and fruits [20]. As a polyphagous species, recorded from at least 242 different host species in 49 different families, *A. lucorum* has been found to switch intensively between habitats and host plants over time [20], [23], [24]. As early as 1958, *A. lucorum* were reported to track locally available flowering plants over the course of a cropping season [25]. Lu et al. [22] found that *A. lucorum* adults preferred cotton plants over other major host crops in mid- to late June in northern China, and proposed that this was because cotton is one of the few flowering host crops locally present during this period. However, much remains to be investigated regarding the plant flower preference of polyphagous *A. lucorum* and the associated ecological mechanisms.

In this study, we related *A. lucorum* adult abundance of on a given plant species with plant phenology data. Our objectives were (1) to assess temporal differences in the extent of flower preference by *A. lucorum* adults, and (2) to assess the role of flower preference as the driver of *A. lucorum* host plant switching.

## Materials and Methods

### Ethics Statement

No specific permits were required for the described field studies.

### Field Trials

During 2007–2012, field studies were conducted at the Langfang Experiment Station of the Chinese Academy of Agricultural Sciences (CAAS, 39.53 °N, 116.70 °E) in Hebei Province of China. For our trials, we planted 131 species of host plants in 2007, 76 species in 2008, 108 species in 2009, 75 species in 2010, 62 species in 2011 and 88 species in 2012, adding up to 174 distinct plant species from 39 families (Table 1), including wild and cultivated plants commonly found in agro-ecosystems of northern China. These 174 species of plant species comprised 74.7% (174 of 233) of the known *A. lucorum* summer host plants. Each plant species was established in three separate 4×4 m plots, with all plots arranged randomly and separated by a 1 m space that was kept free of vegetation by hand weeding. Plots were embedded within a >5 ha cotton field. Plots were established in early May each year through direct seeding and managed using identical agronomic practices among years, while refraining from all insecticide use [26]. Wild plants that were not available commercially as seeds were transplanted as seedlings from nearby agricultural fields. Wild plant species were identified using regional weed guides [27] or with assistance from CAAS plant taxonomists.

**Table 1:** Host plant species assayed during 2007–2012.

Family	Plant species	2007	2008	2009	2010	2011	2012
Amaranthaceae	*Amaranthus retroflexus* L.	+					
Amaranthaceae	*Achyranthes bidentata* Blume	+	+		+	+	+
Amaranthaceae	*Amaranthus tricolor* L.	+	+	+	+		+
Amaranthaceae	*Amaranthus hypochondriacus* L.		+				+
Amaranthaceae	*Celosia cristata* L.	+	+	+	+	+	
Amaranthaceae	*Amaranthus caudatus* L.			+	+	+	
Amaranthaceae	*Gomphrena globosa* L.	+		+		+	
Apocynaceae	*Catharanthus roseus* (L.) G. Don			+	+		+
Araceae	*Arisaema erubescens* (Wall.) Schott	+	+				
Asclepiadaceae	*Telosma cordata* (Burm. f.) Merr.			+	+		+
Asclepiadaceae	*Cynanchum thesioides* (Freyn) K. Schum.	+					
Asclepiadaceae	*Metaplexis japonica* (Thunb.) Mak.	+					
Balsaminaceae	*Impatiens balsamina* L.	+	+	+	+	+	+
Basellaceae	*Basella rubra* L.	+					
Boraginaceae	*Echium vulgare* L.			+	+		+
Boraginaceae	*Borago officinalis* L.			+	+		+
Boraginaceae	*Lithospermum erythrorhizon* Sieb. et Zucc.	+					
Campanulaceae	*Platycodon grandiflorus* (Jacq.) A. DC.	+					
Capparaceae	*Cleome spinosa* Jacq.		+	+	+		+
Capparaceae	*Cleome gynandra* L.	+	+	+	+	+	
Caryphyllaceae	*Dianthus superbus* L.	+	+	+	+	+	
Chenopodiaceae	*Kochia scoparia* (L.) Schrad.	+			+	+	+
Chenopodiaceae	*Beta vulgaris* L.	+		+	+		+
Chenopodiaceae	*Salsola collina* Pall.	+	+	+	+		
Chenopodiaceae	*Chenopodium glaucum* L.	+					
Chenopodiaceae	*Chenopodium album* L.	+					
Chenopodiaceae	*Chenopodium serotinum* L.	+					
Compositae	*Artemisia argyi* Lévl. et Vant.	+	+	+	+	+	+
Compositae	*Artemisia annua* L.	+	+	+	+	+	+
Compositae	*Helianthus annuus* L.	+	+	+	+	+	+
Compositae	*Artemisia lavandulaefolia* DC.	+	+	+	+	+	+
Compositae	*Artemisia scoparia* Waldst. et Kit.	+	+	+	+	+	+
Compositae	*Cosmos sulphureus* Cav.			+	+	+	+
Compositae	*Achillea millefolium* L.			+		+	+
Compositae	*Ixeris denticulata* (Houtt.) Stebb.	+		+			+
Compositae	*Lactuca sativa* L.	+		+			+
Compositae	*Coreopsis tinctoria* Nutt.			+			+
Compositae	*Rudbeckia hirta* L.			+			+
Compositae	*Calendula officinalis* L.			+			+
Compositae	*Taraxacum brassicaefolium* Kitag.	+					+
Compositae	*Taraxacum mongolicum* Hand.-Mazz.	+					+
Compositae	*Cichorium intybus* L.						+
Compositae	*Sonchus brachyotus* DC.						+
Compositae	*Chrysanthemum coronarium* L.	+	+	+	+	+	
Compositae	*Chrysanthemum paludosum* L.			+	+	+	
Compositae	*Ageratum conyzoides* L.			+	+	+	
Compositae	*Coreopsis basalis* L.			+	+	+	
Compositae	*Tagetes patula* L.			+	+	+	
Compositae	*Pyrethrum cinerariifolium* Trev.	+	+	+		+	
Compositae	*Chamaemelum nobile* (L.) All.			+		+	
Compositae	*Zinnia elegans* Jacq.	+		+	+		
Compositae	*Xanthium sibiricum* Patrin ex Widder	+		+			
Compositae	*Carthamus tinctorius* L.	+	+				
Compositae	*Arctium lappa* L.	+	+				
Compositae	*Heteropappus altaicus* (Willd.) Novopokr.	+					
Compositae	*Cirsium setosum* (Willd.) MB.	+					
Compositae	*Bidens bipinnata* L.	+					
Compositae	*Lactuca indica* L.	+					
Compositae	*Tagetes eracta* L.	+					
Compositae	*Inula japonica* Thunb.	+					
Convolvulaceae	*Ipomoea batatas* Lam.	+		+	+		+
Convolvulaceae	*Convolvulus tricolor* L.			+			+
Convolvulaceae	*Pharbitis nil* (L.) Choisy	+	+				+
Convolvulaceae	*Ipomoea aquatica* Forsk.	+					+
Convolvulaceae	*Pharbitis purpurea* (L.) Voight	+					
Cruciferae	*Raphanus sativus* L.	+		+			+
Cruciferae	*Brassica chinensis* L.			+			+
Cruciferae	*Brassica oleracea* L.	+					+
Cruciferae	*Brassica albograbra* L. H. Bailey	+					+
Cruciferae	*Brassica campestris* L.						+
Cruciferae	*Iberis amara* L.	+		+	+	+	
Cruciferae	*Orychophrapmus violaceus* (L.) O. E. Schulz			+	+	+	
Cruciferae	*Brassica juncea* (L.) Czern. et Coss.	+		+		+	
Cruciferae	*Sinapis alba* L.	+	+	+	+		
Cruciferae	*Isatis indigotica* Fort.	+	+	+			
Cruciferae	*Brassica pekinensis* Rupr.	+					
Cucurbitaceae	*Citrullus lanatus* (Thunb.) Mansfeld	+	+	+	+		+
Cucurbitaceae	*Benincasa hispida* (Thunb.) Cogn.	+	+	+			+
Cucurbitaceae	*Cucumis sativus* L.	+	+	+			+
Cucurbitaceae	*Momordica charantia* L.	+	+	+			+
Cucurbitaceae	*Cucurbita moschata* (Duch.) Poiret	+	+	+			+
Cucurbitaceae	*Luffa cylindrica* (L.) Roem.	+	+	+			+
Cucurbitaceae	*Cucurbita pepo* L.	+	+	+			+
Cucurbitaceae	*Cucumis melo* L.	+	+	+			+
Cucurbitaceae	*Trichosanthes kirilowii* Maxim.	+		+			
Dioscoreaceae	*Dioscorea opposita* Thunb.	+	+				
Euphorbiaceae	*Ricinus communis* L.	+	+	+	+	+	+
Euphorbiaceae	*Euphorbia marginata* Pursh.			+			+
Euphorbiaceae	*Acalypha australis* L.	+					
Gramineae	*Sorghum vulgare* Pers.	+	+	+	+	+	+
Gramineae	*Zea mays* L.			+	+	+	+
Gramineae	*Setaria italica* (L.) Beauv.	+	+	+	+		+
Gramineae	*Sorghum sudanense* (Piper) Stapf			+	+	+	
Gramineae	*Coix lacryma-jobi* L.	+	+	+			
Gramineae	*Leptochloa chinensis* (L.) Nees.	+	+				
Labiatae	*Agastache rugosus* (Fisch. et Meyer) O. kuntze.	+	+	+	+	+	+
Labiatae	*Ocimum basilicum* L.	+	+	+	+	+	+
Labiatae	*Leonurus heterophyllus* Sweet	+	+	+	+	+	+
Labiatae	*Salvia farinacea* Benth.		+	+	+	+	+
Labiatae	*Mentha haplocalyx* Briq.	+	+	+	+	+	
Labiatae	*Schizonepeta tenuifolia* (Benth.) Briq.	+	+	+	+	+	
Labiatae	*Scutellaria baicalensis* Georgi	+	+		+	+	
Labiatae	*Hyssopus officinalis* L.	+				+	
Labiatae	*Marjoraan hortensis* Moenoh. syn. Origanum		+	+			
Labiatae	*Salvia officinalis* L.	+					
Labiatae	*Leonurus sibiricus* L.	+					
Labiatae	*Salvia splendens* Ker-Gawler	+					
Leguminosae	*Lablab purpureus* (L.) Sweet	+	+	+	+	+	+
Leguminosae	*Astragalus adsurgens* Pall.	+	+	+	+	+	+
Leguminosae	*Vigna unguiculata* (L.) Walp.	+	+	+	+	+	+
Leguminosae	*Vigna radiata* (L.) Wilczek	+	+	+	+	+	+
Leguminosae	*Phaseolus vulgaris* L.	+	+	+	+	+	+
Leguminosae	*Arachis hypogaea* L.	+	+	+	+	+	+
Leguminosae	*Glycine max* (L.) Merr.	+	+	+	+	+	+
Leguminosae	*Medicago sativa* L.		+	+	+	+	+
Leguminosae	*Onobrychis viciifolia* Scop.		+		+	+	+
Leguminosae	*Astragalus complanatus* Bunge	+	+	+	+		+
Leguminosae	*Mimosa pudica* L.			+			+
Leguminosae	*Melilotus suaveolens* Ledeb.	+	+	+	+	+	
Leguminosae	*Phaseolus coccineus* L.			+	+	+	
Leguminosae	*Vigna angularis* (Willd.) Ohwi et Ohashi	+	+	+	+		
Leguminosae	*Glycyrrhiza uralensis* Fisch.	+	+		+		
Leguminosae	*Trifolium repens* L.	+	+	+			
Leguminosae	*Pisum sativum* L.	+		+			
Leguminosae	*Dolichos lablab* L.	+	+				
Leguminosae	*Trifolium pratense* L.	+	+				
Leguminosae	*Sophora flavescens* Ait.	+	+				
Leguminosae	*Cassia occidentalis* L.	+	+				
Leguminosae	*Coronilla varia* L.	+	+				
Leguminosae	*Cassia tora* L.	+					
Leguminosae	*Vicia villosa* Roth	+					
Liliaceae	*Allium fistulosum* L.	+		+			
Liliaceae	*Allium tuberosum* Rottl. ex Spreng.	+	+				
Linaceae	*Linum usitatissimum* L.	+	+		+	+	
Malvaceae	*Gossypium hirsutum* L.	+	+	+	+	+	+
Malvaceae	*Abutilon theophrasti* Medic.	+		+	+	+	+
Malvaceae	*Althaea rosea* (L.) Cavan.			+	+		+
Malvaceae	*Hibiscus cannabinus* L.	+	+				+
Malvaceae	*Malva sinensis* Cavan.					+	
Malvaceae	*Malope trifida* L.			+	+		
Malvaceae	*Hibiscus esulentus* L.	+		+			
Moraceae	*Cannabis sativa* L.	+	+	+	+	+	+
Moraceae	*Humulus scandens* (Lour.) Merr.	+	+	+	+	+	+
Nyctaginaceae	*Mirabilis jalapa* L.			+	+		
Oleaceae	*Forsythia suspensa* (Thunb.) Vahl	+	+	+			
Onagraceae	*Oenothera odorata* Jacq.			+	+	+	
Pedaliaceae	*Sesamum indicum* L.	+		+	+	+	+
Phytolaccaeae	*Phytolacca acinosa* Roxb.	+					
Polemoniaceae	*Phlox drummondii* Hook.			+			+
Polygonaceae	*Fagopyrum esculentum* Moench	+	+	+	+	+	+
Polygonaceae	*Polygonum orientale* L.	+				+	+
Polygonaceae	*Rheum officinale* Baill.	+					
Portulacaceae	*Portulaca grandiflora* Hook.			+	+	+	+
Ranunculaceae	*Nigella damascena* L.			+			+
Rubiaceae	*Ixora chinensis* Lam.	+					
Rutaceae	*Murraya paniculat* (L.) Jack.			+	+		+
Solanaceae	*Solanum tuberosum* L.	+		+	+		+
Solanaceae	*Nicotiana tabacum* L.			+	+		+
Solanaceae	*Lycopersicon esculentum* Mill.	+		+			+
Solanaceae	*Capsicum annuum* L.	+		+			+
Solanaceae	*Solanum melongena* L.	+		+			+
Solanaceae	*Datura metel* L.	+	+				+
Solanaceae	*Petunia hybrida* Vilm.	+		+			
Solanaceae	*Physalis alkekengi* L.	+		+			
Solanaceae	*Solanum nigrum* L.	+					
Tiliaceae	*Corchorus capsularis* L.	+	+				+
Umbelliferae	*Daucus carota* L. var. sativa DC.			+	+	+	+
Umbelliferae	*Coriandrum sativum* L.	+	+		+	+	+
Umbelliferae	*Apium graveolens* L.	+		+			+
Umbelliferae	*Cnidium monnieri* (L.) Cuss.	+				+	
Umbelliferae	*Saposhnikovia divaricata* (Turcz.) Schischk.	+	+				
Umbelliferae	*Bupleurum falcatum* L.	+	+				
Umbelliferae	*Angelica dahurica* (Fisch. ex Hoffm.) Benth. et Hook. f.	+					
Zygophyllaceae	*Tribulus terrester* L.	+					+

**Note:**+indicates that this plant species was tested in that year. A blank space means no assay.

Each year, we surveyed *A. lucorum* adult abundance within each field plot every 4–5 days from early July to mid-September, coinciding with times of high *A. lucorum* abundance in local agro-ecosystems [20]. Sampling consisted of visually inspecting plants for the presence of *A. lucorum* adults, complemented by knock-down techniques [26]. Both sampling tactics were directed to the upper parts of plants. Knock-down techniques consisted of holding a single plant over a rectangular 40×26×11 cm white-colored pan, and striking it four times, after which the number of dislodged individuals was counted. During each sampling event, we determined the number of *A. lucorum* adults with both sampling methods, and subsequently identified individuals based upon morphological features [28]. Four 1×1 m subplots were sampled within each plot. At each sampling event, we also recorded plant growth stage and presence of flowers for each plant species [22], [26]. For a given plant species, sampling was restricted to times when live plant material was present.

### Data Analysis

A chi-square test was performed to compare the extent to which *A. lucorum* adults visited flowering vs. non-flowering plants during a given specific 2-wk sampling window per year. Each sampling period comprised three or four field surveys. If flowers were found at one or more surveys, the plant species was regarded as “flowering” for the corresponding period. On the other hand, if no flowers were found during any of the surveys, the respective plant species was treated as “non-flowering”.

We calculated the standard attraction (*A*) of a given plant species (*p*) to *A. lucorum* adults at a given sampling date as *Ap* = Pp*n, where *Pp* is relative attraction, defined as the percent abundance of *A. lucorum* adults on plant species *p* versus total adult abundance on all tested plant species, and *n* is a standardization factor, defined as the total number of plant species found with *A. lucorum* adults at the same date [22]. This algorithm eliminates the potential influence of temporal differences in *A. lucorum* population density and number or type of plant species tested between seasons in estimating degree of attractiveness to *A. lucorum* adults of a given plant at a specific sampling date. Each year, we analyzed the most important host plant of *A. lucorum*, cotton (*Gossypium hirsutum* L.) and all other host species with higher adult abundances (i.e., seasonal mean density) than cotton. Standard attraction data for a flowering or non-flowering plant at a given sampling date were considered as replicates in the analysis. Per year, statistical differences in standard attraction between flowering and non-flowering stages for each plant species were determined using analysis of variance (ANOVA) followed by Tukey’s honestly significant differences (HSD) test after verifying the assumptions of normality, homogeneity of variance, and independence. All statistical analyses were performed using SAS/STAT, version 9.1 (SAS Institute, Inc., Cary, NC).

## Results

Over the course of the experiment, the proportion of flowering plants with the presence of *A. lucorum* adults was significantly higher than that of non-flowering plants in each of the different periods (inc. early July, late July, early August, late August, and early September) (*P*<0.05) (Table 2). More specifically, the proportions of flowering and non-flowering plants exploited by *A. lucorum* adults were 50.0–100.0% and 11.3–31.8% in early July, 48.7–95.8% and 10.1–58.3% in late July, 63.6–98.4% and 4.8–51.7% in early August, 71.0–96.4% and 10.9–45.0% in late August, and 73.9–96.3% and 18.2–63.2% in early September, respectively (Table 2).

**Table 2:** The use of flowering and non-flowering host plants by *Apolygus lucorum* adults during different periods from 2007–2012.

Years	Periods	Proportion of flowering plants with the presence of adults (%)	Proportion of non-floweringplants with the presenceof adults (%)	Statistical results of Chi-square analysis
2007	Early July	91.67 (22/24)	31.78 (34/107)	*X^2^* = 28.73; df = 1; *P*<0.0001
	Late July	95.83 (69/72)	47.46 (28/59)	*X^2^* = 39.49; df = 1; *P*<0.0001
	Early August	84.95 (79/93)	27.03 (10/37)	*X^2^* = 41.12; df = 1; *P*<0.0001
	Late August	85.06 (74/87)	26.32 (10/38)	*X^2^* = 41.40; df = 1; *P*<0.0001
	Early September	73.91 (34/46)	30.88 (21/68)	*X^2^* = 20.35; df = 1; *P*<0.0001
2008	Early July	80.00 (8/10)	27.27 (18/66)	*X^2^* = 10.73; df = 1; *P* = 0.0011
	Late July	82.50 (33/40)	58.33 (21/36)	*X^2^* = 5.38; df = 1; *P* = 0.0204
	Early August	90.74 (49/54)	45.45 (10/22)	*X^2^* = 18.46; df = 1; *P*<0.0001
	Late August	96.36 (53/55)	45.00 (9/20)	*X^2^* = 27.00; df = 1; *P*<0.0001
	Early September	91.30 (42/46)	48.15 (13/27)	*X^2^* = 17.06; df = 1; *P*<0.0001
2009	Early July	100.00 (11/11)	11.34 (11/97)	*X^2^* = 47.88; df = 1; *P*<0.0001
	Late July	48.72 (19/39)	10.14 (7/69)	*X^2^* = 20.28; df = 1; *P*<0.0001
	Early August	63.64 (42/66)	4.76 (2/42)	*X^2^* = 36.85; df = 1; *P*<0.0001
	Late August	71.01 (49/69)	13.89 (5/36)	*X^2^* = 30.91; df = 1; *P*<0.0001
	Early September	83.33 (20/24)	18.18 (14/77)	*X^2^* = 34.78; df = 1; *P*<0.0001
2010	Early July	88.89 (24/27)	22.92 (11/48)	*X^2^* = 30.22; df = 1; *P*<0.0001
	Late July	62.26 (33/53)	22.73 (5/22)	*X^2^* = 9.72; df = 1; *P* = 0.00182
	Early August	98.44 (63/64)	36.36 (4/11)	*X^2^* = 37.96; df = 1; *P*<0.0001
	Late August	94.23 (49/52)	27.27 (6/22)	*X^2^* = 36.32; df = 1; *P*<0.0001
	Early September	96.30 (26/27)	70.73 (29/41)	*X^2^* = 6.88; df = 1; *P* = 0.0087
2011	Early July	66.67 (22/33)	24.14 (7/29)	*X^2^* = 11.21; df = 1; *P*<0.0001
	Late July	80.95 (34/42)	55.00 (11/20)	*X^2^* = 4.59; df = 1; *P* = 0.0323
	Early August	93.33 (42/45)	41.18 (7/17)	*X^2^* = 20.26; df = 1; *P*<0.0001
	Late August	90.70 (39/43)	36.84 (7/19)	*X^2^* = 19.96; df = 1; *P*<0.0001
	Early September	95.83 (23/24)	63.16 (24/38)	*X^2^* = 8.56; df = 1; *P* = 0.0034
2012	Early July	50.00 (12/24)	14.06 (9/64)	*X^2^* = 12.41; df = 1; *P* = 0.0004
	Late July	70.37 (38/54)	23.53 (8/34)	*X^2^* = 18.35; df = 1; *P*<0.0001
	Early August	81.36 (48/59)	51.72 (15/29)	*X^2^* = 8.39; df = 1; *P* = 0.0038
	Late August	83.05 (49/59)	34.48 (10/29)	*X^2^* = 20.76; df = 1; *P*<0.0001
	Early September	79.63 (43/54)	27.27 (9/33)	*X^2^* = 23.35; df = 1; *P*<0.0001

**Note:** Data in parentheses represent the number of plant species with the presence of *A. lucorum* adults and the total number of plant species at flowering or non-flowering stages, respectively.

**Figure 1:**
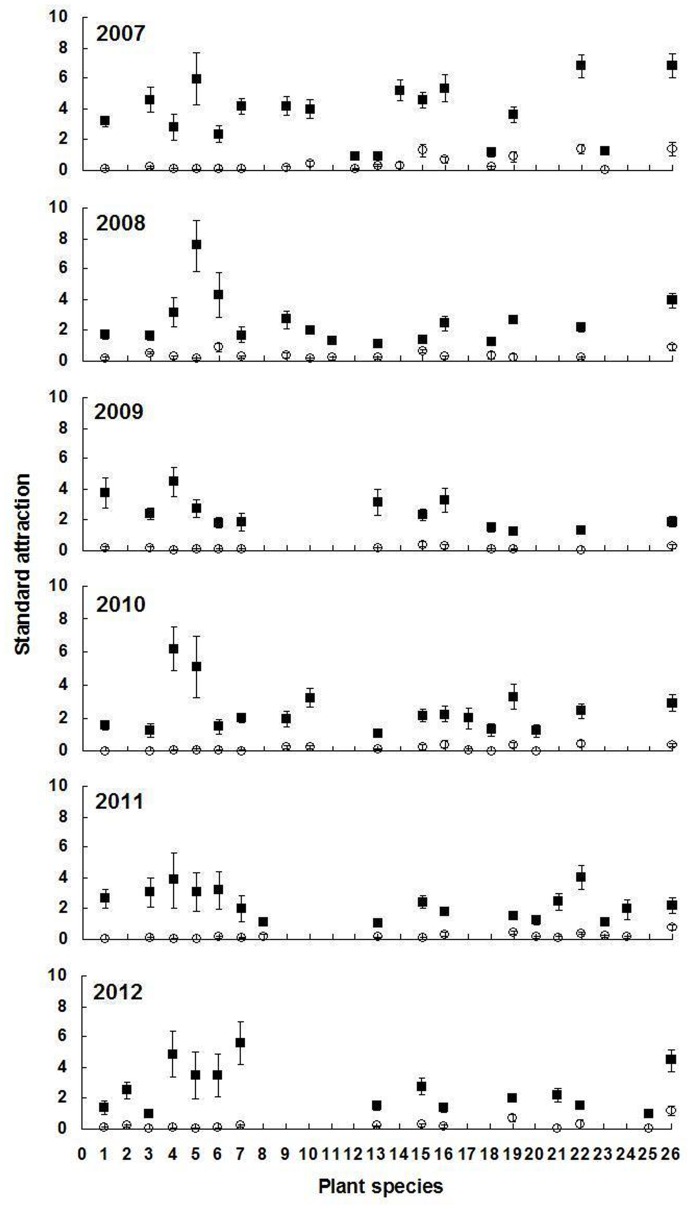
Standard attraction of different host plants during flowering (black diamonds) and non-flowering (grey dots) periods for *Apolygus lucorum* adults from 2007–2012. Means (±SE) between flowering and non-flowering periods are significantly different for each plant species per year (*P*<0.05). The blank indicates no assay. Plant species: 1 *Agastache rugosus* (Fisch. et Meyer) O. kuntze., 2 *Amaranthus hypochondriacus* L., 3 *Artemisia annua* L., 4 *Artemisia argyi* Lévl. et Vant., 5 *Artemisia lavandulaefolia* DC., 6 *Artemisia scoparia* Waldst. et Kit., 7 *Cannabis sativa* L., 8 *Chamaemelum nobile* (L.) All., 9 *Chrysanthemum coronarium* L., 10 *Coriandrum sativum* L., 11 *Dianthus superbus* L., 12 *Fagopyrum esculentum* Moench, 13 *Gossypium hirsutum* L., 14 *Helianthus annuus* L., 15 *Humulus scandens* (Lour.) Merr., 16 *Impatiens balsamina* L., 17 *Linum usitatissimum* L., 18 *Mentha haplocalyx* Briq., 19 *Ocimum basilicum* L., 20 *Oenothera odorata* Jacq., 21 *Polygonum orientale* L., 22 *Ricinus communis* L., 23 *Schizonepeta tenuifolia* (Benth.) Briq., 24 *Sorghum vulgare* Pers., 25 *Telosma cordata* (Burm. f.) Merr., 26 *Vigna radiata* (L.) Wilczek.

**Figure 2:**
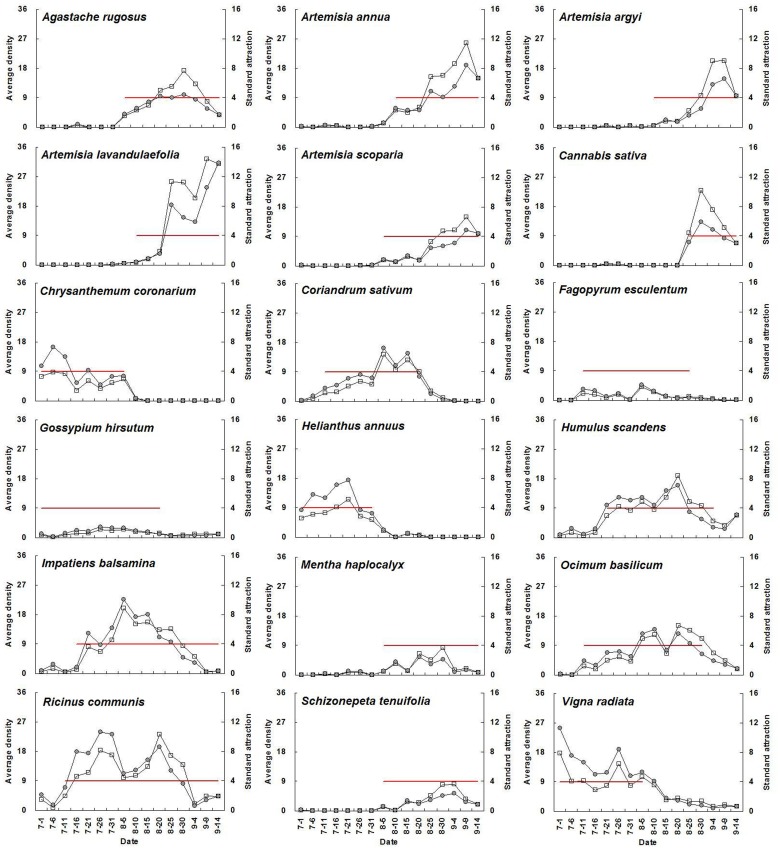
Seasonal changes of population density of *Apolygus lucorum* adults and standard attraction of each host plant during 2007. The red line indicates the flowering period. Data of population dynamics of *A. lucorum* on cotton (*Gossypium hirsutum* L.) and mungbean (*Vigna radiata* (L.) Wilczek) in 2007 were cited from [26].

**Figure 3:**
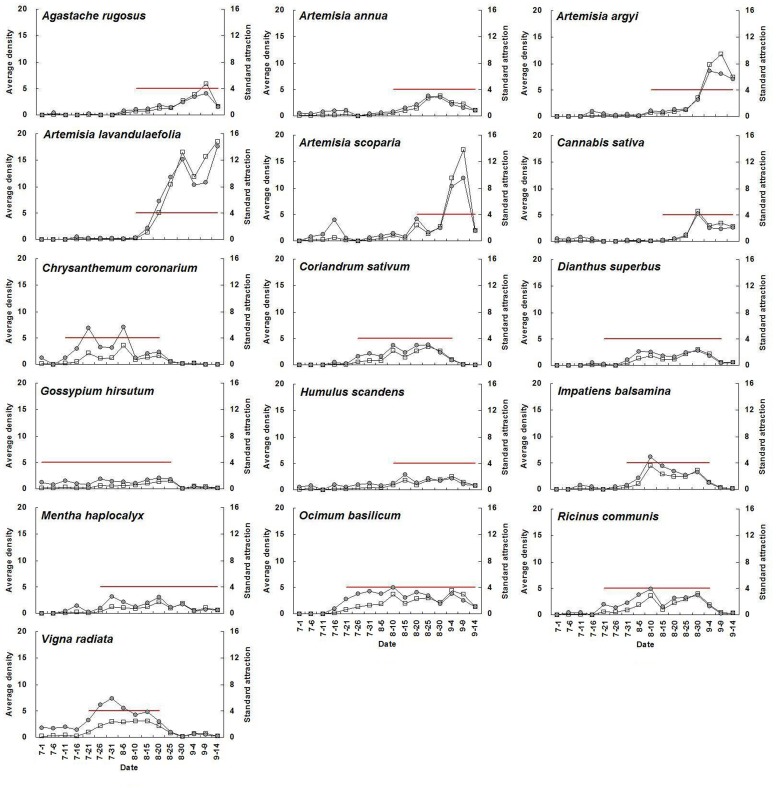
Seasonal changes of population density of *Apolygus lucorum* adults and standard attraction of each host plant during 2008.

**Figure 4:**
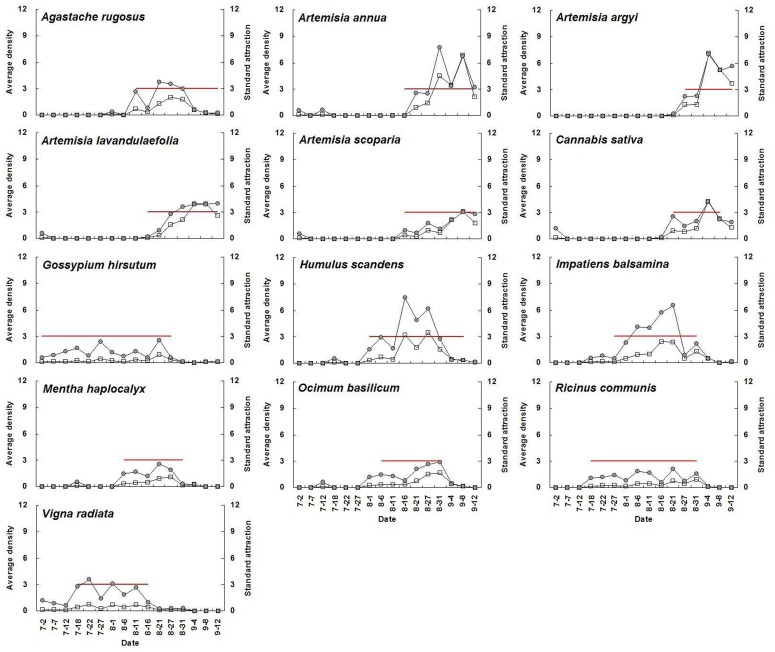
Seasonal changes of population density of *Apolygus lucorum* adults and standard attraction of each host plant during 2009.

**Figure 5:**
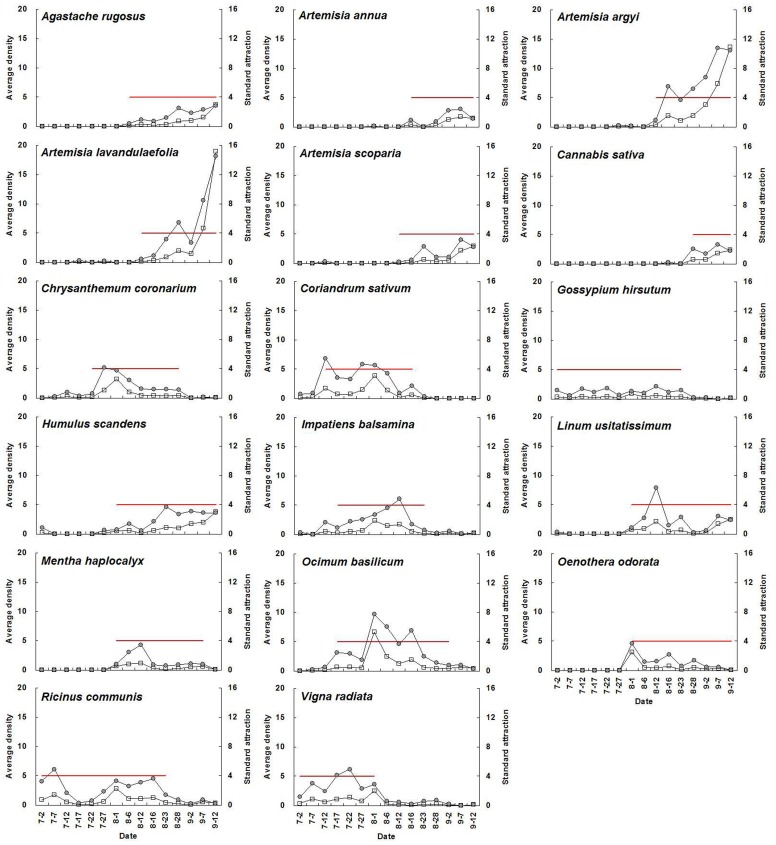
Seasonal changes of population density of *Apolygus lucorum* adults and standard attraction of each host plant during 2010.

**Figure 6:**
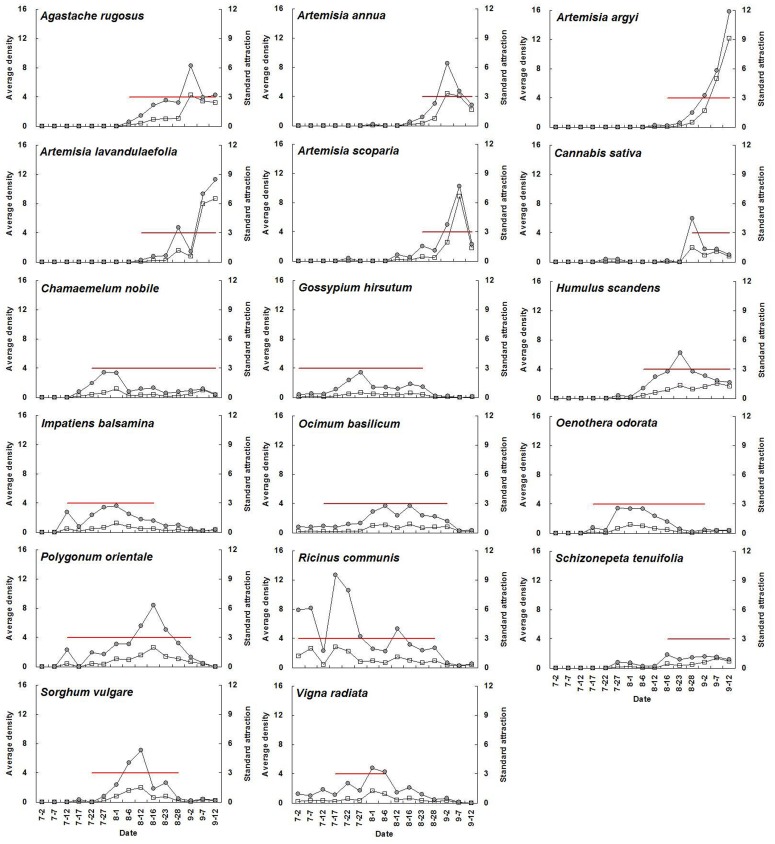
Seasonal changes of population density of *Apolygus lucorum* adults and standard attraction of each host plant during 2011.

**Figure 7:**
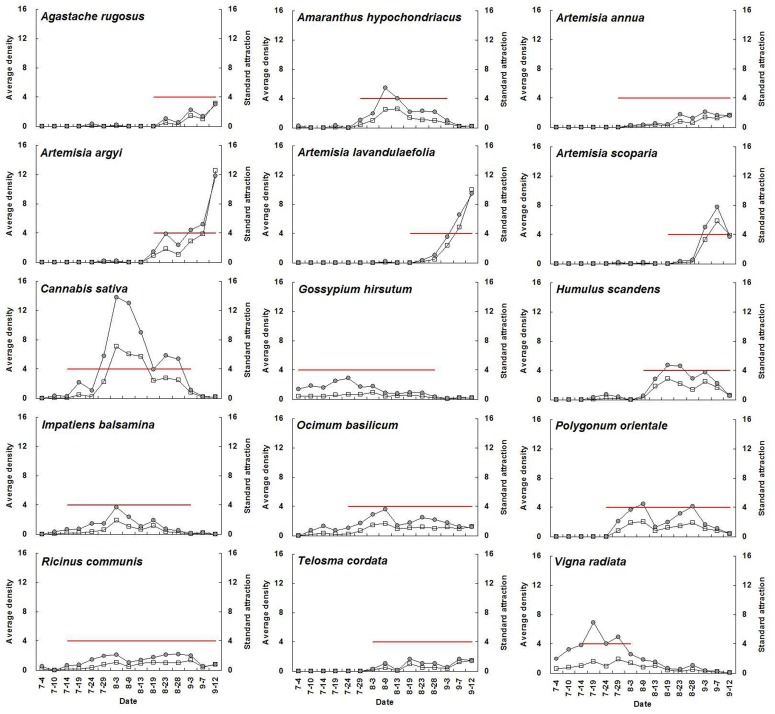
Seasonal changes of population density of *Apolygus lucorum* adults and standard attraction of each host plant during 2012.

For a given plant species with high adult abundance, standard attraction during flowering periods was significantly higher than during non-flowering periods (*P*<0.05) (Figure 1, Table 3). The average standard attraction of all selected flowering plants at flowering stage was 9.3, 7.7, 19.5, 15.5, 12.9, and 12.3 times higher than that during non-flowering periods from 2007 until 2012, respectively. Seasonal fluctuations in *A. lucorum* adult abundance on each plant species and the relative standard attraction for a given plant species showed similar trends. The mean population level of the above plant species at flowering stage was 10.3, 17.8, 28.9, 18.6, 13.9, and 18.2 times higher than that during non-flowering periods from 2007 to 2012, respectively (Figure 2–7).

**Table 3:** Comparison of the standard attraction of each plant species at flowering and non-flowering periods for *Apolygus lucorum* adults during 2007–2012.

No.	Plant species	2007	2008	2009	2010	2011	2012
1	*Agastache rugosus* (Fisch. et Meyer) O. kuntze.	*F* = 60.38; df = 1,14; *P*<0.0001	*F* = 23.25; df = 1,14; *P* = 0.0003	*F* = 11.08; df = 1,14; *P* = 0.0050	*F* = 21.69; df = 1,13; *P* = 0.0004	*F* = 16.56; df = 1,13; *P* = 0.0013	*F* = 12.80; df = 1,13; *P* = 0.0034
2	*Amaranthus hypochondriacus* L.						*F* = 17.02; df = 1,13; *P* = 0.0012
3	*Artemisia annua* L.	*F* = 31.88; df = 1,14; *P* = 0.0001	*F* = 12.80; df = 1,14; *P* = 0.0030	*F* = 16.60; df = 1,14; *P* = 0.0011	*F* = 16.30; df = 1,13; *P* = 0.0014	*F* = 21.50; df = 1,13; *P* = 0.0005	*F* = 8.41; df = 1,13; *P* = 0.0124
4	*Artemisia argyi* Lévl. et Vant.	*F* = 10.34; df = 1,14; *P* = 0.0062	*F* = 9.27; df = 1,14; *P* = 0.0087	*F* = 52.52; df = 1,14; *P*<0.0001	*F* = 24.82; df = 1,13; *P* = 0.0003	*F* = 6.91; df = 1,13; *P* = 0.0209	*F* = 16.05; df = 1,13; *P* = 0.0015
5	*Artemisia lavandulaefolia* DC.	*F* = 11.91; df = 1,14; *P* = 0.0039	*F* = 19.43; df = 1,14; *P* = 0.0006	*F* = 26.72; df = 1,14; *P* = 0.0001	*F* = 8.29; df = 1,13; *P* = 0.0129	*F* = 6.73; df = 1,13; *P* = 0.0223	*F* = 7.75; df = 1,13; *P* = 0.0155
6	*Artemisia scoparia* Waldst. et Kit.	*F* = 14.50; df = 1,14; *P* = 0.0019	*F* = 8.61; df = 1,14; *P* = 0.0109	*F* = 31.11; df = 1,14; *P* = 0.0001	*F* = 13.10; df = 1,13; *P* = 0.0031	*F* = 13.16; df = 1,13; *P* = 0.0031	*F* = 3.72; df = 1,13; *P* = 0.0758
7	*Cannabis sativa* L.	*F* = 136.18; df = 1,14; *P*<0.0001	*F* = 9.84; df = 1,14; *P* = 0.0073	*F* = 47.12; df = 1,14; *P*<0.0001	*F* = 150.10; df = 1,13; *P*<0.0001	*F* = 15.42; df = 1,13; *P* = 0.0017	*F* = 5.14; df = 1,13; *P* = 0.0410
8	*Chamaemelum nobile* (L.) All.					*F* = 5.48; df = 1,13; *P* = 0.0359	
9	*Chrysanthemum coronarium* L.	*F* = 14.88; df = 1,9; *P* = 0.0039	*F* = 8.64; df = 1,12; *P* = 0.0124		*F* = 11.07; df = 1,13; *P* = 0.0054		
10	*Coriandrum sativum* L.	*F* = 17.18; df = 1,12; *P* = 0.0014	*F* = 33.00; df = 1,14; *P* = 0.0001		*F* = 23.71; df = 1,13; *P* = 0.0003		
11	*Dianthus superbus* L.		*F* = 8.66; df = 1,14; *P* = 0.0107				
12	*Fagopyrum esculentum* Moench	*F* = 10.25; df = 1,14; *P* = 0.0064					
13	*Gossypium hirsutum* L.	*F* = 9.09; df = 1,14; *P* = 0.0093	*F* = 28.52; df = 1,14; *P* = 0.0001	*F* = 10.34; df = 1,14; *P* = 0.0062	*F* = 22.70; df = 1,13; *P* = 0.0004	*F* = 7.99; df = 1,13; *P* = 0.0143	*F* = 8.57; df = 1,13; *P* = 0.0118
14	*Helianthus annuus* L.	*F* = 44.40; df = 1,11; *P*<0.0001					
15	*Humulus scandens* (Lour.) Merr.	*F* = 18.45; df = 1,14; *P* = 0.0007	*F* = 10.59; df = 1,14; *P* = 0.0058	*F* = 9.86; df = 1,14; *P* = 0.0072	*F* = 15.01; df = 1,13; *P* = 0.0019	*F* = 31.62; df = 1,13; *P* = 0.0001	*F* = 16.69; df = 1,13; *P* = 0.0013
16	*Impatiens balsamina* L.	*F* = 16.54; df = 1,14; *P* = 0.0012	*F* = 19.82; df = 1,14; *P* = 0.0005	*F* = 15.07; df = 1,14; *P* = 0.0017	*F* = 10.00; df = 1,13; *P* = 0.0075	*F* = 24.36; df = 1,13; *P* = 0.0003	*F* = 5.08; df = 1,13; *P* = 0.0422
17	*Linum usitatissimum* L.				*F* = 6.67; df = 1,13; *P* = 0.0228		
18	*Mentha haplocalyx* Briq.	*F* = 9.53; df = 1,14; *P* = 0.0080	*F* = 5.87; df = 1,14; *P* = 0.0295	*F* = 33.69; df = 1,14; *P*<0.0001	*F* = 10.58; df = 1,13; *P* = 0.0063		
19	*Ocimum basilicum* L.	*F* = 12.97; df = 1,14; *P* = 0.0029	*F* = 30.31; df = 1,14; *P* = 0.0001	*F* = 29.66; df = 1,14; *P* = 0.0001	*F* = 7.48; df = 1,13; *P* = 0.0170	*F* = 8.16; df = 1,13; *P* = 0.0135	*F* = 8.44; df = 1,13; *P* = 0.0123
20	*Oenothera odorata* Jacq.				*F* = 7.38; df = 1,13; *P* = 0.0176	*F* = 5.83; df = 1,13; *P* = 0.0313	
21	*Polygonum orientale* L.					*F* = 6.76; df = 1,13; *P* = 0.0220	*F* = 8.07; df = 1,13; *P* = 0.00139
22	*Ricinus communis* L.	*F* = 21.69; df = 1,14; *P* = 0.0004	*F* = 27.15; df = 1,14; *P* = 0.0001	*F* = 37.69; df = 1,14; *P*<0.0001	*F* = 7.53; df = 1,13; *P* = 0.0168	*F* = 5.16; df = 1,13; *P* = 0.0407	*F* = 6.47; df = 1,13; *P* = 0.0245
23	*Schizonepeta tenuifolia* (Benth.) Briq.	*F* = 20.10; df = 1,14; *P* = 0.0005				*F* = 59.22; df = 1,13; *P*<0.0001	
24	*Sorghum vulgare* Pers.					*F* = 6.67; df = 1,13; *P* = 0.0227	
25	*Telosma cordata* (Burm. f.) Merr.						*F* = 18.38; df = 1,13; *P* = 0.0009
26	*Vigna radiata* (L.) Wilczek	*F* = 36.90; df = 1,14; *P*<0.0001	*F* = 43.53; df = 1,14; *P*<0.0001	*F* = 31.14; df = 1,14; *P* = 0.0001	*F* = 30.54; df = 1,13; *P* = 0.0001	*F* = 10.89; df = 1,13; *P* = 0.0058	*F* = 24.54; df = 1,13; *P* = 0.0003

**Note:** A blank space means no assay.

The use of flowering plant species by *A. lucorum* adults varied during the course of the sampling period. In early July, *A. lucorum* adults preferred a small number of species, such as *Vigna radiata* (L.) Wilczek., *Gossypium hirsutum* L., *Helianthus annuus* L. and *Chrysanthemum coronarium* L., which were in flower. In late July, adults dispersed more widely into other hosts (e.g. *Ricinus communis* L., *Impatiens balsamina* L., *Humulus scandens* (Lour.) Merr., *Ocimum basilicum* L., *Agastache rugosus* (Fisch. et Meyer) O. kuntze. and *Coriandrum sativum* L.), and usually maintained high population levels through August. In early September, *A. lucorum* largely migrated to blooming *Artemisia* spp. (e.g. *A. argyi* Lévl. et Vant., *A. lavandulaefolia* DC., *A. annua* L. and *A. scoparia* Waldst. et Kit.) (Figure 2–7).

## Discussion

In earlier work, seasonal host switching of certain polyphagous mirid bugs (e.g. *L. lineolaris*, *Pseudatomoscelis seriatus* [Reuter]) has been related to their preference for flowering host plants [19], [29], [30]. In our study, *A. lucorum* equally exhibited a clear preference for flowering plants and switched food plants according to the succession of different flowering plant species in the local agro-ecosystem [22], [25]. It provided important information for further understanding the interaction between *A. lucorum* and host plants, and exploring the patterns of population dynamics of this mirid bug in different host plants.

The polyphagous species *A. lucorum* prefers to feed on tender leaves, buds and flowers, which usually become scarce after flowering stage [20]. To locate suitable food, *A. lucorum* adults exhibit a clear preference for flowering plant species in the process of host plant switching. This strategy of host plant switching helps offset seasonal or year-to-year changes in host abundance [31] and also allows mirid bugs to avoid intra- and interspecific competition for host plants. In 2010, *I. balsamina* plants were badly infected with powdery mildew in early August, making those plants less suitable for *A. lucorum* population growth. As a result, most adults dispersed to other host plants and the abundance in *I. balsamina* decreased dramatically. Similar population dynamics were also found in other host plants with serious pest infestations during the study, supporting our speculation that *A. lucorum* altered host plants primarily to find suitable food.

Through host plant switching hemimetabolous insects, such as mirid bugs, possibly can increase their population growth [32]. For example, *L. lineolaris* shows different rates of reproduction on different hosts, and host switching thus can considerably increase its population growth and survival [33]. In a laboratory study, *A. lucorum* adults and nymphs had higher survival and fitness on mungbean (*V. radiata*) over cotton [34], and on flowering individuals of three plant species (*G. hirsutum*, *R. communis* and *I. balsamina*) [32]. However, it is unknown which parts of the flowers (e.g., pollen, flower nectars) are the main food sources for *A. lucorum* or which nutrients (e.g., sugars, amino acids) are the most important for the increase of its population fitness [32]. Additionally, *A. lucorum* preference-performance relationship for flowering plants needs to be assessed in field conditions, as other ecological factors such as natural enemy abundance, environmental conditions, and broader host plant availability can affect host plant choice [35].

At a given time, *A. lucorum* showed a clear preference for a limited number of plants species. As not all plant species are present in all agricultural landscapes of northern China, *A. lucorum* abundance is deemed highly dependent upon location and composition of local agricultural landscapes [36]. In China, there are different cropping patterns, including mixed plantations of food crops and cotton, fruit trees and cotton, pastures and cotton, and so forth [37]. In each cropping pattern, the dominant overwintering location and seasonal host plant range of *A. lucorum* vary considerably [24], which would lead to different patterns of host plant use (inc. seasonal dynamics, between-plant transfer).

Our work showed year-by-year fluctuations in general *A. lucorum* abundance (Figure 2–7), which affected its population levels on a given host plant at any specific time. Yearly differences in climatic conditions and associated plant germination and growth are thought to be the prime determinants of those seasonal patterns [32], [38], [39]. Computer models maybe help to simulate its population dynamics in the agro-ecosystem and then analyze the effects of various biotic factors (e.g., host plant selection, phenological relative survival) and abiotic factors (e.g. temperature, rainfall) on its seasonal occurrence [40].

For many phytophagous insects, host switching is guided by host plant volatiles [41], [42]. Adults of *A. lucorum* are attracted to variable extent to different plant species in Y-tube olfactometer trials [43], with electro-antennogram (EAG) responses to (E)-2-hexenal and other plant volatiles [44]. Increase in *A. lucorum* abundance on flowering plants may hint that adults orient themselves to specific volatiles of flowering plants. Visual cues may further enhance their behavioral response to plant volatiles [45]. However, for *A. lucorum* as for many other mirid bugs, much remains to be learned about the exact chemical and non-chemical determinants of flower preference.

Recently, there has been increasing interest in the application of behavioral manipulation methods (e.g. trap cropping) as a component of integrated pest management (IPM) strategies [46], [47], [48], [49]. Our elucidation of considerable variation in *A. lucorum* abundance among host plants and among different periods (Figure 2–7), will contribute to the development of sustainable management strategies for *A. lucorum*. Previous work has led to the use of *V. radiata* as a trap crop for *A. lucorum* in Bt cotton fields [26]. This work also provides several other potential trap plants of *A. lucorum* and aids in identify the attractive volatile compositions, all of which could be developed as new alternative methods of controlling this mirid bug [49], [50].

Agricultural landscapes dominated by crops and uncultivated habitats may contribute in increasing or decreasing pest population density in the fields, therefore analyzing the temporal variability of source and sink effects is of importance for managing the placement of landscapes to promote pest control. For example, Ting [51] found that the population abundance of mirid bug complex (mainly including *A. lucorum*, and *Adelphocoris suturalis* (Jakovlev), *Adelphocoris lineolatus* (Goeze), *Adelphocoris fasciaticollis* (Reuter)) in alfalfa fields at middle April were positively correlative with that in cotton field at early July. Carrière et al. [2] reported that abundance of seed alfalfa and cotton flowering date were positively associated with *Lygus* density in cotton fields, whereas abundances of cotton and uncultivated habitats were negatively associated with *Lygus* density in cotton. Our present study provide an ability to explore the source/sink role of different plant species as factors affecting population dynamics of *A. lucorum*, and aiding the development of landscape-level pest management strategies.
